# Patient’s and health care provider’s perspectives on music therapy in palliative care – an integrative review

**DOI:** 10.1186/s12904-018-0286-4

**Published:** 2018-02-20

**Authors:** W. Schmid, J. H. Rosland, S. von Hofacker, I. Hunskår, F. Bruvik

**Affiliations:** 1 GAMUT (Grieg Academy Research Centre for Music Therapy) Faculty of Fine Arts, Music and Design, University of Bergen, Bergen, Norway; 2 Sunniva Centre for Palliative Care, Haraldsplass Deaconess Hospital, Bergen, Norway; 3 Department of Clincal Medicine, University of Bergen, Bergen, Norway; 4 Regional Centre of Excellence for Palliative Care Western Norway, Haukeland University Hospital, Bergen, Norway; 5 VID Specialized University, Bergen, Norway; 6 Haraldsplass Deaconess Hospital, Bergen, Norway; 7 Department of Global Public Health and Primary Care, Centre for Elderly and Nursing Home Medicine, University of Bergen, Bergen, Norway

**Keywords:** Music therapy, Palliative care, Patient reported outcomes, Interventions, Pain, Physical comfort

## Abstract

**Background:**

The use of music as therapy in multidisciplinary end-of-life care dates back to the 1970s and nowadays music therapy (MT) is one of the most frequently used complementary therapy in in-patient palliative care in the US. However existing research investigated music therapy’s potential impact mainly from *one* perspective, referring to either a quantitative or qualitative paradigm. The aim of this review is to provide an overview of the users’ and providers’ perspectives on music therapy in palliative care within one research article.

**Methods:**

A systematic literature search was conducted using several databases supplemented with a hand-search of journals between November 1978 and December 2016. Inclusion criteria were: Music therapy with adults in palliative care conducted by a certified music therapist. Both quantitative and qualitative studies in English, German or a Scandinavian language published in peer reviewed journals were included. We aimed to identify and discuss the perspectives of both patients and health care providers on music therapy’s impact in palliative care to forward a comprehensive understanding of it’s effectiveness, benefits and limitations. We investigated themes mentioned by patients within qualitative studies, as well as commonly chosen outcome measures in quantitative research. A qualitative approach utilizing inductive content analysis was carried out to analyze and categorize the data.

**Results:**

Twelve articles, reporting on nine quantitative and three qualitative research studies were included. Seven out of the nine quantitative studies investigated pain as an outcome. All of the included quantitative studies reported positive effects of the music therapy. Patients themselves associated MT with the expression of positive as well as challenging emotions and increased well-being. An overarching theme in both types of research is a psycho-physiological change through music therapy.

**Conclusions:**

Both quantitative as well as qualitative research showed positive changes in psycho-physiological well-being. The integration of the users´ and providers´ perspectives within future research applicable for example in mixed-methods designs is recommended.

## Background

Individuals with incurable diseases and limited life expectancy are vulnerable and often in need for multidisciplinary palliative care. This care should address the physical, emotional, social, and spiritual needs of an individual, applying a patient-centered approach. Within this holistic approach the therapeutic use of music has become increasingly implemented [1]. The use of music as therapy in multidisciplinary end-of-life care dates back to the 1970s [2]. Nowadays music therapy (MT) is one of the most frequently used complementary therapy in palliative care in the US [3], and has been widely implemented internationally within the last decades in this area [4].

In music therapy, patient and therapist engage actively in singing, songwriting, improvisation, as well as listening to music, according to a person’s musical preferences ([5]. Within a therapeutic relationship based on individualized assessment, treatment and evaluation individual and situative music experiences can evolve [6].

Music therapy applies a wide range of elaborated approaches, enloys high acceptance by patients and has few side effects [1, 3]. However existing research investigates music therapy’s potential impact mainly from *one* perspective, referring either to a quantitative or qualitative paradigm.

Individual needs as well as the possible variety of MT approaches at hand add to the ethical and methodological complexity for research. It has been suggested that the inclusion of diverse research paradigms, allowing for multiple ways of knowing and forms of evidence, might be more purposeful [7]. Furthermore, patients’ perspectives have been widely acknowledged as being important for the definition of outcome measures as well as to cast light on mechanisms of therapeutic change [8]. This is also in line with the recommendations of the WHO-paper “Vision in people-centered health care”, addressing the future culture of care and communication, and advocating for the involvement of health care users in decision-making [9]. Refering to qualitative research, patient’s perspective add essential evidence to music therapy’s contribution in palliative care and can inform future research. However this evidence needs to be integrated more systematically. To our knowledge, no comprehensive research discussing both patient’s and health care services’ perspectives more systematically has been published so far. One way of approaching this is the conduction of an integrative review. An integrative review is a specific review method allowing for the assessment of diverse data sources and methodologies such as qualitative interviews or standardized questionnaires [10]. Integrative reviews have the potential to present a comprehensive understanding of phenomena or problems relevant to health care and policy. They present the state of the art, and can contribute to theory development [11].

We have been able to identify only one integrative review with the focus of MT in palliative care [12]. According to this review, music is a positive stimulus to improve coping for patients at the end of life. However, this conclusion was drawn wihout including the patients´ perspective.

To address this gap of knowledge, the aim of this integrative review is to identify and discuss the perspectives of both patients and health care providers on music therapy’s impact in palliative care. With this integration of multiple ways of knowing, the intention is to reach a more comprehensive understanding of what domains are sensitive to change in music therapy with the terminally ill.

## Methods

An integrative approach conducted by an interdisciplinary team of researchers and practitioners located at Sunniva Center for Palliative Care (Haraldsplass Deaconess Hospital, Bergen, Norway) and The Grieg Academy Research Centre for Music Therapy (GAMUT, University of Bergen, Norway).

To ensure a clear focus, a transparent, comprehensive collection and extraction of data, and to handle the complexity inherent in combining diverse methodologies we followed the five stages of an integrative review as formulated by Whittemore & Knafl [10]: problem identification, literature search, data evaluation, data analysis and presentation.

### Literature search

On the basis of a Cochrane systematic review [13] and a state-of-the-art article about the subject [14] a preliminary search was conducted early in June 2015. The research group discussed the research questions, and which search terms and databases to include.

The main literature search was conducted in June 2015 within the databases MEDLINE, AMED, CINAHL, EMBASE, PsychInfo, OVID Nursing, RILM, Web of Science and in the Nordic databases NORART (Norwegian articles) and SweMed+ (Nordic Health articles) (Table 1). The first publication about music therapy in palliative care was also included at this stage [2].

**Table 1:** Search strategy

1. palliative care/ or terminal care/ or hospice care/ or terminally ill/	
2. palliative care.mp. or exp. Palliative Care/	
3. terminal care.mp. or Terminal Care/	
4. exp. Hospice Care/	
5. Hospice Care.mp. or Hospice Care/	
6. exp. Terminally Ill/	
7. terminally ill.mp. or Terminally Ill/	
8. hospice*.tw.	
9. (palliat* or (terminal* adj6 ill*) or (terminal* adj3 care) or (end adj3 life)).tw.	
10. ((care adj5 dying) or (caring adj5 dying) or (support$ adj5 dying) or (dying adj5 patient$)).tw.	
11. 1 or 2 or 3 or 4 or 5 or 6 or 7 or 8 or 9 or 10	
12. music therapy.mp. or exp. Music Therapy/	
13. music*.mp.	
14. melody.mp.	
15. (music$ or melod$).tw.	
16. (sing or sings or singer$ or singing or song$).tw.	
17. 12 or 13 or 14 or 15 or 16	
18. 11 and 17	
19. protocol*.tw.	
20. 18 not 19	
21. limit 18 to yr. = “1978 -Current”	

We included quantitative, qualitative and mixed methods research studies on music therapy with a trained music therapist in a palliative care setting including adult in- and out-patients (Table 5). The main outcomes were both observed symptoms and self-reported experiences. Peer reviewed publications in English, German or Scandinavian languages were included. Study protocols, feasibility-studies as well as single case studies were excluded. The inclusion and exclusion criteria as well as the search terms were worked out by the PICO model according to population, intervention, comparison and outcome [15].

An updated search was conducted in December 2016 using the same search strategy in the same databases limited to the year 2014 and forward. A total of 233 articles were found and scanned by the researchers. One article was included in the analysis after the updated search.

We applied standardized evaluation schemes for all types of studies (RCT-studies, quantitative and qualitative studies) using the CASPs checklists [16, 17]. The CASP’s checklists are widely used in the health care domaine and offer guidance for the critical appraisal with respect to trustworthiness, results and relevance of research studies. Criteria for the quality assessment of RCT’s are, e.g. the critical appraisal of a study’s validity and treatment effect. Criteria for the quality assessment of qualitative studies are, e.g. the appraisal of a clearly stated aim, an appropriate qualitative methodology, and the consideration of ethical issues. The quality check was conducted by two researchers independently. Qualitative studies that scored at least 7 out of ten of the criteria in the CASP checklist, and quantitative studies that scored at least 8 out of 11 of the criteria in the CASP checklist were included for further analysis.

## Results

**Fig. 1:**
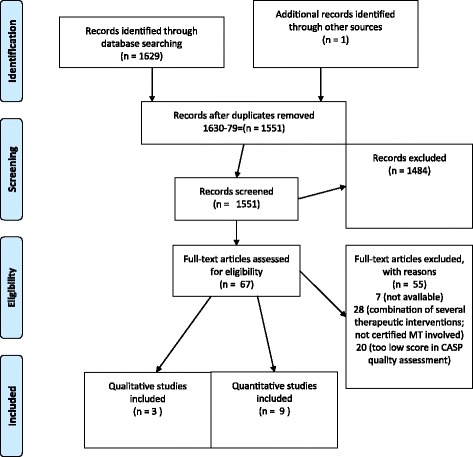
PRISMA Flow Diagram (attached)

A total of 1629 articles was identified (Fig. 1). One article could be included into the sample after hand-searching. A manual duplicate control removed 79 publications, leaving 1551 articles for further evaluation. Altogether 1484 articles were excluded, including six review articles. Reference lists from these six review articles were scanned for relevant publications “meeting the inclusion criteria and not yet included”. No further articles were found in this step of the process.

Altogether 67 studies were assessed for eligibility and read in full-text in pairs. In the case of disagreement we discussed in the research group until a consensus was reached. As part of this phase, we conducted a quality-appraisal with all studies to be included, using the CASPs checklists [16, 17]. After completion of the screening and evaluation, twelve articles were left to be included in this review, reporting on nine quantitative and three qualitative research studies. No mixed-methods studies were found.

In a next step, we extracted data on frequency, and duration of the music therapy intervention, and whether the intervention was applied individually or in a group setting. In addition we extracted the outcomes and patient’s reports (Tables 2 and 3). The main outcomes were observed and self-reported experiences and symptoms.

**Table 2:** Quantitative studies

Author	Participants	Intervention	Control	Results
Design RCT				
Warth, et al. [3](Germany)	*N* = 84 Mean age 63 Inpatient	MTs × 2 Not patient-centered MT	Listened to a verbal relaxation exercise	Subjective improved relaxation, well-being and fatigue-subscale. Increase in high-frequency oscillations of the heart rate. MT was not found to contribute to acute pain reduction.
Gutgsell, et al. [21] (USA)	*N* = 200 Mean age 56 Inpatient	MTs × 1, therapist-guided relaxation	Relax no instructions	Decline of pain
Clements-Cortes [34] (Canada)	*N* = 40 Age 40–95 Inpatient	MTs Individualized	Individualized taped MTs	Pain reduction and enhancement of physical comfort.
Horne-Thompson & Grocke, [22](Australia)	*N* = 25 Age 18–90 Inpatient	MTs ×1 Individualized active and receptive	Volunteer visit	Reduction in anxiety, pain, tiredness and drowsiness. No significant effect in a decrease in heart rate.
Hilliard, [24] (USA)	*N* = 80 Mean age 65 Outpatient	MTs × 2 (− 13) individualized	TAU	Improved QoL No significant differences on functional status or length of life.
Pre-Post Design				
Domingo et al. [19] (Spain)	*N* = 68 Mean age 73 Inpatient	MTs × 4 in group individualized music/songwriting	TAU Not by random	Effect emotional distress and well-being. No significant effect of pain observed.
Nakayama et al. [20] (Japan)	*N* = 10 Mean age 73 Inpatient	MT Small group with mainly receptive method.	No	Lowering of salivary cortisol levels Decreased symptoms of anxiety and depression. No change in fatigue levels
Gallagher et al. [23] (USA)	N = 200 24–87 years Inpatient	MTs × 1 individualized	No	Improvements in anxiety, body movement, facial expression, mood, pain, shortness of breath, and verbalizations.
Krout, [39] (USA)	N = 80 Age 38–97 In/out patient	MTs × 1 individualized active and receptive	No	Effect in observed and self-reported pain control, physical comfort, and relaxation.

*MTs* Music therapy session, *TAU* treatment as usual

**Table 3:** Qualitative studies

Author	Design	Participants	Intervention	Results: Participant’s experiences categorized in themes
Clements-Cortes, [34](Canada)	Cross-case analysis; Thematic analysis	4 individuals 63–91 years Inpatient	Patient- centered Individualized MT (24–35 sessions), 14–20 weeks	(1) love, (2) loss, (3) gratitude, (4) growth/transformation, (5) courage/strength, and (6) good-bye.
O’Callaghan, [25] (Australia)	Grounded Theory; thematic analysis with ATLAS.ti	128 individuals 16–101 years Inpatient	At least one patient centered MTs in individual and group setting.	MT can elicit (1) varied affective responses, (2) shifts in physical awareness, (3) rediscovered or new self-awareness. Music can be associated with (4) experiencing altered or improved awareness, (5) increased well-being, (6) human relationships, (7) or does “nothing” to some.
Teut, M. et al., [26] (Germany)	Grounded Theory; thematic analysis with MAXQDA	8 individuals 51–82 years Inpatient	Up to 5 individual MT sessions weekly. Focus on somatic listening applying a Body Tambura.	(1) Relaxing and calming effects, (2) sensations that the body feels lighter, and (3) the provocation of peaceful images or visualizations.

*MT* Music therapy

A qualitative approach utilizing inductive content analysis was carried out to analyze and categorize the data in the three qualitative studies [18]. Two members of the research group identified, compared and organized the categories into thematic clusters.

### Quantitative studies

Nine quantitative studies (Table 2) published between 2001 and 2016 were included. Four of these studies origined from the USA and one from Canada; two studies were conducted in Europe (Spain and Germany), one is from Australia and another one from Japan. Five of the studies were RCTs, four had a pre-post research design with measurements taking place before and after the music therapy intervention. For the RCT studies the participants were randomized to either intervention or control group receiving standard care or an extra intervention as: Volunteer visit, individualized taped MT, relaxation without instructions and listening to a verbal relaxtion exercise. In two of the RCTs, a computer program was used for study randomization, one study describes a numbered envelope prosess, and two of the studies did not describe the randomization prosess. Also one of the studies with a pre-post design had a non randomized control group with TAU. Three of the studies did not have a control group. Seven studies were conducted in hospital-based inpatient palliative care units, one at privat homes, and one study covered both, in- and out-patient settings. The studies included between 10 and 200 participants (63% female) from ages 18 to101 years old, all diagnosed with a terminal illness such as cancer, COPD, AIDS, ALS or other neurodegenerative diseases. Apart from the fact that study-participants were cared for in settings commonly associated with terminal illness (as in-patients in a hospice, on a palliative care ward or receiving palliative care home services), the term “terminal illness” was not defined more specifically in the included studies.

All studies provided live music as part of the MT intervention. In five of the nine studies music therapy was provided in individual one-to-one settings taking patients´ music preferences as the starting point. Two studies [19, 20] conducted MT in small groups. In two of the RCTs a standardized music therapy program was applied, comprised of a combination of musical exercises and relaxation [3], or autogenic training [21]. The numbers of sessions conducted in the studies varied from 1 to 13 sessions, with four studies conducting only one session.

The most commonly investigated outcome in the quantitative studies was pain. Both observer rated and patient rated outcomes were reported, applying standardized questionnaires and measurement tools as well as VAS and behavioral scales.

Seven out of the nine studies measured pain and pain perception as main outcome, with five of the seven studies reporting a decrease of pain after music therapy (Table 2). In six of the seven studies pain was a main outcome. In the study of Horne-Thompson and Grocke [22], pain was a secondary outcome. The RCT of Warth and colleagues [3] did not find an effect of a standardized music based relaxation exercise on pain reduction. Domingo and colleagues et al. [19] did not find any significant improvement for the outcome pain in their study. However, evaluation of pain was not carried out pre and post single sessions, but at the very end of a 7 days intervention period.

Next to pain, well-being and mood was most often recorded. Positive effects on well-being were reported in two studies [3, 19] and mood (including depression, anxiety) was reported to improve in four studies [19, 20, 23]. Hilliard [24] found in his study that Quality of life improved for patients receiving MT, and the effect increased over time as they had more sessions. However, Quality of life as well as relaxation and fatigue did not show clearly positive or negative results in the studies (Table 4). These findings are in line with research conducted earlier [4, 12]. Nakayama and colleagues [20] employed the salivary cortisol level to measure stress in participants, and found significant lowering of levels after music therapy session.

**Table 4:** Outcome quantitative studies

Outcome	Effect	Study
Well being (VAS)	Effect	Domingo et al. [19]
Emotional distress (HADS)	Effect	Domingo et al. [19]
Pain and asthensia (sub scale)	No differances between groups	Domingo et al. [19]
Acute Pain (SR-VAR)	No differances between groups	Warth et al. [3]
Well-being (VAS-SR)	Effect	Warth et al. [3]
Relaxation, ((VAS) SR)	Effect	Warth et al. [3]
Heart rate variability	Effect	Warth et al. [3]
Health related quality of life (QLQ-C15-PAL)	No difference between groups	Warth et al. [3]
QoL – Fatigue (QLQ-C15-PAL)	Effect	Warth et al. [3]
The FLACC Scale (pain observation)	No differences between groups	Gutgsell et al. [21]
Numeric rating scale pain (SR)	Effect	Gutgsell et al. [21]
The Functional Pain Scale (SR interview)	Effect	Gutgsell et al. [21]
Present Pain Intensity	No differences between groups	Clements-Cortes [34]
McGill Pain questionnaire	No differences between groups	Clements-Cortes [34]
Physical comfort (VAS SR)	No differences between groups	Clements-Cortes [34]
Pain perception (VAS- SR)	No differences between groups	Clements-Cortes [34]
S-cotisol level	Effect	Nakayama et al. [20]
The Mood Inventory Scale -Fatigue	No effect	Nakayama et al. [20]
The Mood Inventory Scale -refreshment (SR)	Effect	Nakayama et al. [20]
The Mood Inventory Scale -anxiety/ depression	Effect	Nakayama et al. [20]
ESAS Anxiety (SR)	Effect	Horne-Thompson & Grocke [22]
Pulse oximeter for heartrate	No differences between groups	Horne-Thompson & Grocke [22]
ESAS Tiredness, drowsiness, pain (SR)	Effect	Horne-Thompson& Grocke [22]
ESAS Nausea, depression, appetite, well-being, Shortness of breath	No differences between groups	Horne-Thompson & Grocke [22]
Shortness of breath (VAS)	Effect	Gallagher et al. [23]
Mood, depression, anxiety, (VAS-SR)	Effect	Gallagher et al. [23]
Pain (VAS)	Effect	Gallagher et al., [23]
Facial, movement and verbal (by therapist)	Effect	Gallagher et al. [23]
Length of life	No differences between groups	Hilliard [24]
Hospice QoL -functional well-being (SR)	No differences between groups	Hilliard [24]
Hospice QoL –psychophysiological well-being	Effect	Hilliard [24]
Hospice QoL -social/spiritual	No differences between groups	Hilliard [24]
Palliative Performance Scale (spl R)	No differences between groups	Hilliard [24]
Pain control (observed SR)	Effect	Krout [39]
Relaxation (observed SR)	Effect	Krout [39]
Physical comfort (observed SR)	Effect	Krout [39]

Having a closer look on the instruments and methods used for the measurement of pain in the nine studies, six applied patient-reported scales (VAS), two collected data from both patients and observers (i.e. nurses or research assistants), and one operated with observer’s data only. From the six studies based on patient’s reports, five described positive effects on pain after one or two sessions music therapy. In four of these studies individualized music therapy based on patients’ preferences was offered.

### Qualitative studies

Three qualitative studies (Table 3) published between 2001 and 2014 reporting research conducted in Canada, Australia and Germany were included in this review. Studies’ designs embrace qualitative interviews and thematic analyses in a cross-case study design or adapted Grounded Theory [25, 26]. All three studies investigated individual music therapy in inpatient individual- or group-settings. The studies included 4–128 participants (50% female) between 16 and 101 years of age, diagnosed with advanced cancer of the lungs, the pancreas or brain tumor. None of the studies defines “terminal illness” explicitly. The number of sessions (1–35 sessions) varied greatly between the studies, as well as MT methods offered. In two of the three studies music therapy followed the participant’s individual preference and daily form. Accordingly, the therapist offered music listening, songwriting, instrumental improvisation and musical life reviews [24, 25]. In the study of Teut and colleagues [26] a standardized music therapy program with a Body Tambura, a wooden string instrument that is placed on or close to the human body, was applied.

All qualitative studies presented and categorized experiences of MT from the patient’s perspective. Applying an inductive content analysis, the categories were grouped to three main clusters [18]. The clusters are presented in the following, referring to categories as presented in the three studies:

Patient’s themselves associated music therapy with

1. the expression of both, positive as well as more challenging emotions (referring to categories: *love; loss; transformation; strength; ambivalent emotions*)
2. a relaxing and calming effect with shifts in physical awareness and increased well-being (*feelings of relaxation; shifts in physical awareness; increased well-being and self-awareness*)
3. addressing relational issues like loss and saying goodbye, love, or gratitude to family and close friends (*connecting to family; relationships; memories; self-expression*).

Next to these benefits for the individual, family members and friends who could be present in the MT sessions, sing or listen to familiar music with their loved ones, felt more connected with the patient, and found support for grieving processes [25, 26].

## Discussion

The studies assembled in this integrative review report a range of benefits and positive effects of music therapy in palliative care from both the patient’s as well as the health care provider’s perspective. All the included quantitative studies reported several positive effects of MT. Four studies found significant pain reduction after only one session of individualized music therapy. However, two other studies could not demonstrate any effect on pain and pain perception [3, 19]. Although no definite explanation can be given for this, the methodology chosen in the studies by Warth et al. [3], and Domingo and colleagues [19] could be crucial. It is remarkable that the study by Warth and colleagues [3] utilized a standardized music based relaxation exercise, meaning that individual music preferences were not taken into consideration as the starting point for the MT intervention. In light of an ongoing discussion about the potential relevance of patient-preferred music in MT, a number of studies including a recent meta-analysis [27], do note that music must be tailored appropriately to individual preferences to realize the greatest benefit from the intervention [27]. Another aspect that needs to be taken into account is the point of time of measurement. Domingo and colleagues [19] did not evaluate pain directly pre and post single sessions, but after the last session, that is after 7 days. As pain perception is a dynamic process, it could be modified, and perhaps exacerbated by many other factors, from day 1 to day 7, such as disease progression or changes in medication inversely interfering with the expected effect of the music therapy intervention.

The overarching theme promoted in both types of research is a psycho-physiological change through music therapy. While patients report a reduction of pain in the quantitative studies, they rarely mention specific symptoms like pain in the qualitative studies. However, they report improvements of physical comfort with changes in bodily awareness, emotional relief and positive relationship experiences on intra- and interpersonal levels.

As both pain and well-being are core issues being addressed in end-of-life care, it could be of interest to explore potential interconnections between the reduction of pain on one side, and improvement of physical comfort and well-being on the other side.

Pain is subjective, highly complicated in nature, and may be exhibited very differently from individual to individual depending on their physical as well as psychological state [31]. There is growing acknowledgement of pain as one of the most significant challenges to well-being, with a potential to impact considerably an individuals quality of life [28–30]. Research suggests that pain can become resistant to conventional treatment measures if psychological, emotional, or spiritual issues are not addressed [31, 32].

Music is known to have a wide range of physiological effects on the human body, including changes in heart rate, respiration, blood pressure, and biochemical responses [33]. Musical experience has been described as the “richest human emotional, sensorimotor, and cognitive experience” (ibid, p 12). Responses to music and pain are based on past experiences and/or present state of mind and are highly individual [34]. By altering affective, cognitive and sensory processes, music therapy may reduce pain perception and suffering, heighten mood, and increase a sense of control and relaxation [25].

Furthermore, “a shared positive experience of the music therapy seemed to facilitate a connection between the patients and the family members” [24]. Music therapy that included family members and acknowledges musical preferences of the individuals involved, offers an arena for the facilitation and completion of relationships to oneself and – likewise - to others [35].

When asked of what exactly was experienced while participating in MT, many patients describe relaxing and calming effects, causing sensations that the body feels lighter. They report increased well-being, and the calming and relaxing effect of music: “Well, it (...) feels somehow like swimming on waves, where you feel good” or have feelings of “lightness”, or “as if floating in the air” [26] (p.4). Another participant stated: “I became so absorbed in the music and my aches and pains disappeared” [25] (p.158). As condensed in this last quote, an interconnection of the experience of pain and well-being becomes obvious. In the process of psycho-physiological change, the perception of pain and a reduction of stress seems to correspond with feelings of “lightness”, “floating in the air”, and the experience of sense of control.

On this background, the impact of somatic music experiences on an individuals bodily and physiological situatedness as shown in the study of Teut and colleagues [26] is remarkable. The integration of both vibrations, sounds, and music might facilitate relaxation, and contribute to the reduction of levels of anxiety and stress [36]. As shown by Teut and colleagues [26], a multimodal approach embracing sound and vibration within a therapeutic relationship has a direct impact on an individuals well-being.

In summary music therapy as a relational and experiental based approach, does not work like a medication to reduce a symptom. It is rather an embodied practice embedded in whole body-actions of the individuals involved, and capable to respond to an individual’s needs in an ever changing process [37, 38].

### Limitations of the integrative review

We focused on the outcome pain in the analysis of the quantitatve studies. Pain is one of the most relevant clinical symptoms in palliative care, and we therefore did not differ between pain as main and secondary outcome in our review. We discussed both pain and well-being, representing two prominent phenomena from out of all included studies, to exemplify a potential integration. This can be seen as a limitation of the present review. At the same time it can serve as an example for how the integration of perspectives can enhance a more comprehensive understanding of music therapy’s contribution to end-of-life care. To further investigate other outcomes in this way might be of interest.

Within the included studies, some methodological limitations could be identified with respect to a lack of the definition of “terminal illness”, and incomprehensive provision of information about samples, randomization, drop out rates, as well as the music therapy intervention and setting itself. With respect to the inclusion criteria of our review, the focus was on the qualification of the music therapist. This allowed at the same time for the application of a variety of music therapeutic approaches to be included. In this way we could take into account the need for a variety of approaches that can be flexibly used by a professional to meet individual’s ever changing needs in palliative care.

### Implications for future research

With respect to the symptom pain and corressponding themes mentioned by patient’s themselves, we could show that an integration of perspectives can enhance a more comprehensive understanding of music therapy in end-of-life care. On this background we want to recommend for further research:

1. The implementation of mixed-methods studies where different perspectives and research paradigms can be integrated in *one* study.
2. More frequent measurements and patient feedback, pre-post but also during a music therapy session to further approach the question of music therapy’s effect within the therapeutic course.

## Conclusions

Individual music therapy seems to have positive impact on several symptoms and needs, thus improving individuals´ quality of life in the palliative care setting. The present review contributes to exsting research by systematically integrating patient’s and health care provider’s perspective on music therapy. This leads to an enhanced and comprehensive understanding of what music therapy can contribute to interdisciplinary end-of-life care. The integration of users´ and providers´ perspectives within future research applicable for example in mixed-methods designs is recommended.
